# Exploring the Potential of Epigallocatechin Gallate in Combating Insulin Resistance and Diabetes

**DOI:** 10.3390/nu16244360

**Published:** 2024-12-18

**Authors:** Kübra Yurtseven, Sevinç Yücecan

**Affiliations:** 1 Department of Nutrition and Dietetics, Institute of Health Sciences, Lokman Hekim University, 06510 Çankaya, Ankara, Turkey; 2 Department of Nutrition and Dietetics, Faculty of Health Sciences, Lokman Hekim University, 06510 Çankaya, Ankara, Turkey; sevinc.yucecan@lokmanhekim.edu.tr

**Keywords:** epigallocatechin gallate (EGCG), diabetes, insulin resistance, glycemic control, beta cell function, insulin sensitivity

## Abstract

Background/Objectives: In this study, the potential effects are evaluated of epigallocatechin gallate (EGCG) on the prognosis of diabetes and insulin resistance. Methods: In an experiment, 35 male Wistar albino rats were used and in the streptozotocin (STZ)-induced diabetic rats, the effects were examined of different doses (50 mg/kg, 100 mg/kg, 200 mg/kg) of EGCG on metabolic parameters associated with diabetes and insulin resistance. Results: The findings show favorable effects of EGCG on fasting blood glucose levels, insulin secretion, insulin resistance, and beta cell function. In this study, it was observed that EGCG was able to significantly lower fasting blood glucose levels, especially at high doses (200 mg/kg), providing the most significant improvement. Furthermore, EGCG has been found to reduce insulin resistance and improve insulin sensitivity by increasing insulin secretion. When the biochemical parameters of increased insulin secretion are evaluated, it is also observed that it creates clinically significant changes. At doses of 100 mg/kg and 200 mg/kg, EGCG has the potential to help control diabetes by most effectively improving insulin resistance and beta cell function. The study results suggest that EGCG, especially at high doses, is an effective component in the treatment of diabetes and the management of insulin resistance. Conclusions: The inclusion of EGCG as a natural flavonoid in medical nutrition therapy may contribute to glycemic control and improve insulin sensitivity in individuals with diabetes. These findings suggest that EGCG may be used as an alternative option in the treatment of diabetes and future studies may further clarify the potential benefits in this area.

## 1. Introduction

Diabetes has developed into one of the most critical and prevalent chronic diseases. of our time, leading to life-threatening complications, long-term health problems, and significant healthcare costs, as well as reducing the quality of life and shortening life expectancy [1]. Diabetes is defined by elevated blood glucose levels caused by deficient insulin production or ineffective insulin, and these raised blood glucose levels can damage many organs (such as the eyes and kidneys) by affecting the nervous and circulatory systems [2]. Type 1 (T1D) and type 2 (T2D) diabetes are the most prevalent types of diabetes. T1D occurs when the body’s immune system mistakenly attacks and eliminates the beta cells in the pancreas that produce insulin. In T2D, insulin production is not stopped altogether; instead, insulin either becomes ineffective due to mutations or the body becomes resistant to it, causing its effectiveness to decrease. T2D is increasingly recognized as a global health emergency affecting 9.3% of the world’s population [3]. Gestational diabetes is a disorder of glucose metabolism that occurs for the first time during pregnancy without any previous problems and increases women’s risk of developing T2D later in life [4,5].

The worldwide diabetes rates have grown into a global health emergency with the International Diabetes Federation (IDF) reporting 9% (463 million adults) in 2019, in its 9th edition of its Atlas. The expanding prevalence of diabetes is chiefly driven by the increasing life expectancy. However, important drivers of higher prevalence include the increase in the incidence of diabetes in some countries due to the increasing prevalence of diabetes risk factors, particularly obesity, as well as decreasing mortality among people with diabetes due to improved medical care [6,7]. The prevalence of type 2 diabetes, which accounts for the vast majority of all diabetes cases (almost 90%), has been reported to increase with age and is also associated with increasing urban development and environments that promote unhealthy weight gain [8].

A detailed analysis of the prevalence of diabetes according to the IDF 2019 Atlas reveals that 463 million adults have diabetes, with 374 million affected by impaired glucose tolerance (IGT), with around 80% of these cases occurring in low- and middle-income countries [9]. In the wider European context, the prevalence of diabetes among adults averages at 8.9% and unfortunately affects more than 59 million people. It is thought that 40% of adult diabetics in Europe, more than 24 million people, are undiagnosed [10]. The escalating impact of diabetes, driven by factors such as poor dietary habits, obesity, physical inactivity, and genetic predispositions, poses a major challenge to health systems worldwide. In 2021, the global prevalence of impaired glucose tolerance in adults aged 20–79 years was approximately 9.1% (464 million), and this number is expected to reach an estimated 638 million by 2045, an increase of 10% [11].

Given the wide array of signaling pathways and complex molecular mechanisms involved in the development of insulin resistance (IR), a much deeper understanding of the underlying processes is essential to identify novel, comprehensive therapeutic approaches to prevent and manage this complex condition. In clinical terms, the term “insulin resistance” refers to the need for high levels of insulin to maintain normal glucose concentrations. At the molecular level, insulin resistance is characterized by inefficient insulin signal transduction in pathways from the insulin receptor (INSR) to the final targets of insulin. This leads to impaired cellular function and metabolic processes as well as intercellular communication [12]. Glucose homeostasis is primarily regulated by the secretion of insulin from beta cells in response to elevated blood glucose levels following food intake. The effectiveness of this regulation depends on the insulin sensitivity of key tissues, including the liver, skeletal muscle, and adipose tissue. A highly coordinated insulin-mediated signaling pathway governs a range of metabolic functions, such as reducing hepatic glucose production, enhancing glucose uptake in muscle and fat cells, and inhibiting the release of free fatty acids (FFAs) from adipocytes. These insulin actions are finely tuned to ensure the optimal metabolic activity and maintain energy homeostasis [13]. Insulin resistance (IR), which arises from disruptions in any of these processes and the failure of target cells to adequately respond to insulin, involves both intracellular and extracellular signaling disturbances [14]. Insulin resistance is strongly affected by nutrient overload because nutrient overload results in the activation of inflammatory signaling cascades, endoplasmic reticulum (ER) stress and mitochondrial dysfunction, and the accumulation of metabolic by-products in the tissues responsible for insulin recognition [15].

Antidiabetic drugs are recommended primarily and frequently in the management of diabetes. However, antidiabetic drugs have side effects (gastrointestinal disorders, weight loss or gain, hypoglycemia attacks, etc.) [16]. The side effects of these drugs make the search for natural substances an attractive and potential alternative way to prevent or treat T2D. Among the most widely consumed flavonoids, flavan-3-ols have been associated with various health benefits, including the prevention of chronic diseases (such as cardiovascular disease and cancer). Epidemiologic studies on type 2 diabetes (T2D) suggest that foods rich in flavan-3-ols, especially epigallocatechin gallate (EGCG) found in green tea, may help reduce the risk of developing diabetes [17]. EGCG is the predominant polyphenol in green tea, accounting for more than 50% of its total polyphenolic composition, known as catechins, and numerous scientific studies have shown that EGCG is responsible for many of the potential health benefits ascribed to green tea [18]. EGCG has been reported to have a wide range of potential health-promoting effects such as reducing inflammation, preventing cancer, combating obesity, inhibiting microbial growth, fighting infections, protecting the nervous system, and modulating immune responses [19,20,21,22,23].

Possible molecular mechanisms that may assist in curbing or managing diabetes include improving glucose uptake in muscle and adipose tissue through activation of the insulin signaling pathway as well as increasing beta cell function and survival, enhancing incretin action, and reducing endogenous glucose production. When other mechanisms on the antidiabetic effects of EGCG are examined, the suppression of oxidative stress, correction of endothelial dysfunction, modulation of cytokine expression, modulation of gene expression, and the direct antihyperglycemic effects of EGCG can be listed [17].

Our study was planned to evaluate the possible antidiabetic effects of EGCG consumption in light of the mechanisms described in these research results in the literature. Unlike the studies in the literature, EGCG intake was not provided intraperitoneally in our study plan, but EGCG was given orogastrically to the rats in order to evaluate more objectively and accurately in terms of nutrition. Thus, in line with our study results, it will be more effective to evaluate the antidiabetic effects of green tea consumption in daily diet [17].

## 2. Materials and Methods

### 2.1. Animal Model and DM Induction

All experiments performed in this study were approved by the Research Ethics Committee of Kobay Experimental Animals Laboratory, Turkey (Decision number: 677 Acceptance date: 21 July 2023). In this study, 35 male, 8-week-old Wistar albino rats weighing 250–300 g obtained from the Kobay Experimental Animal Laboratory were used. All rats were laboratory-reared and evaluated by veterinarians who regularly checked their health status. The sample size was decided by G*power analysis. All rats were selected from the same breed (Wistar albino) and housed in cages with optimum laboratory conditions (23 ± 1 °C, 55 ± 5% humidity, 12 h light/dark cycle) (after the rats were divided into groups, the groups were housed in the same cages), continuous feeding (pellet-based feed specially produced for small laboratory animals), and fresh water 7 days before the beginning of the experiment and throughout the experimental period.

Rats in the groups in which diabetes was to be induced (rats in the other groups except the healthy group) were treated with a single dose of 45 mg/kg streptozotocin (STZ) [24,25]. STZ was dissolved in 0.01 M citrate buffer (pH: 4.5) and administered intraperitoneally. The healthy group was injected with the same amount of saline. Glucose levels in blood samples taken from the tail vein 48 h after STZ injection were determined by PlusMED Accuro brand biosensor glucose meter and strips. Those with blood glucose levels of 220 mg/dL and above were considered diabetic and included in this study. In rats with blood glucose levels below 220 mg/dL, 30 mg/kg STZ [26] administration was repeated and fasting blood glucose measurements were repeated 48 h after the additional dose administration. A total of 28 of 35 rats (except the healthy group) were administered STZ. Since diabetes did not occur in 3 of the STZ-treated rats, an additional dose was needed. Diabetes was detected in the 48th hour measurements of the rats to which the additional dose was administered. 

During the experiment, healthy and adaptable animals were included in this study. Animals that showed aggressive behavior or had health problems related to their health status were not included in this study. While the rats were in the acclimatization process before the experiment, it was found that one rat exhibited aggressive behavior and tried to harm other rats. This rat was removed from this study and a new rat that met the conditions was taken into this study immediately afterwards. In each experimental group, blood was taken for biochemical analyses with animal welfare as the primary concern for analysis. In addition, EGCG treatment was given by oral gavage for 21 days. During all these procedures, they were not harmed in any way and the procedures were carried out professionally. For each analysis, the number of n in each experimental group as well as in the control group and the healthy group was seven (the number of subjects was decided by G*power analysis).

### 2.2. Experimental Design

**Figure 1:**
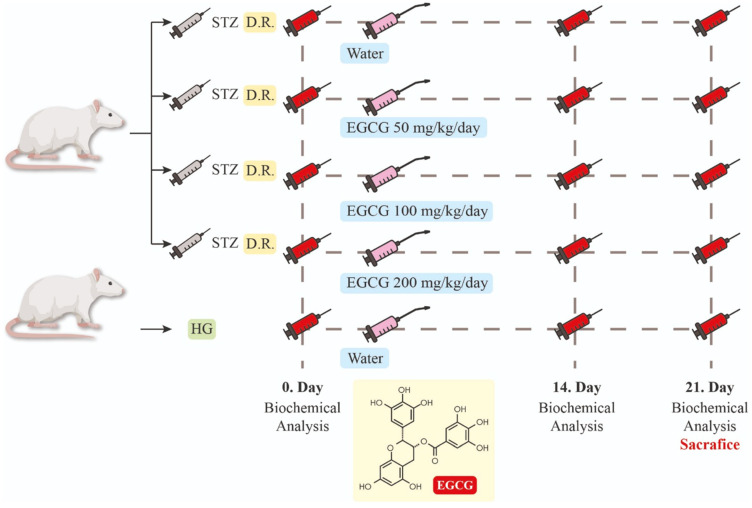
Experimental design. (D.R.; diabetic rats, HG; healthy group).

The subjects were divided into five groups, each consisting of seven rats, as follows: healthy (HG), diabetes-control (D), rats with diabetes induced and given 50 mg EGCG per kg, rats with diabetes induced and given 100 mg EGCG per kg, and rats with diabetes induced and given 200 mg EGCG per kg. The experimental design is summarized in detail in Figure 1. EGCG supplementation was started immediately after diabetes was established in rats by fasting blood glucose assessment. Rats for each group were randomized on the basis of age, weight, and health. The rats requiring additional STZ administration were distributed as follows: 50 mg/kg EGCG group, 100 mg/kg EGCG group, and 200 mg/kg EGCG group. It was checked that there was no difference between the groups in terms of age and weight.

After diabetes was induced in rats, the treatment process was initiated and EGCG powder was diluted with drinking water and given intragastrically at 50 mg/kg, 100 mg/kg, and 200 mg/kg [27,28,29]. The healthy group and the diabetes control group were given only drinking water by oral gavage every day (21 days) during the experimental period as in the EGCG-treated groups. The daily oral gavage administration was performed safely and without stressing the rats in the immediate vicinity of their cages.

Biochemical analyses during the 21-day treatment period, fasting glucose levels, and fasting insulin levels were determined from blood samples obtained from the tail vein of the rats at the beginning (day 0), day 14, and day 21. For fasting biochemical parameters, rats were fasted for 12 h and then blood was collected [30]. Body weights of the rats were recorded during these periods. Handling and sampling during blood collection were performed in a way that minimized stress and ensured animal welfare. As insulin levels and glucose levels are the simple parameters needed to compute HOMA-IR and HOMA-B, they have become the most commonly used markers to assess insulin resistance, beta cell function, and glucose metabolism [31].

In our study, in order to determine insulin resistance, insulin sensitivity, and beta-cell function by using fasting blood glucose and fasting insulin values [32], homeostasis model assessment by formulation-insulin resistance (HOMA-IR) index and quantitative insulin sensitivity control index (QUICKI) were calculated in rats. The HOMA-IR index was calculated using the formulation of fasting insulin level (mUI/l) and fasting glucose level (mg/dL)/405 [33,34]. HOMA insulin sensitivity index (HOMA-IS) was calculated using the formulation = 10,000/[fasting glucose level × fasting insulin level] [35,36]. HOMA-β = [fasting plasma insulin (μIU/mL) × 360/(fasting plasma glucose (mg/dL)-63] [37,38]. It was calculated using the formulation and the QUICKI = 1/[log (insulin μU/mL) + log (glucose mg/dL) [38,39].

At the conclusion of this study, the rats were anesthetized and then sacrificed.

The veterinarian in the laboratory and the principal investigator were aware of the group allocation during the distribution of the rats and the conduct of the experiment. Only the principal investigator was aware of the group allocation during result evaluation and data analysis. When blood samples were analyzed and body weight was measured, it was not specified which sample belonged to which group of rats. The principal investigator was present at all these stages to ensure smooth functioning. There were no unexpected events during this study.

### 2.3. Chemicals and Materials

Streptozotocin (STZ) was purchased from Sigma-Aldrich (S0130-1G, Sigma Chemical Co., St. Louis, MO, USA), epigallocatechin gallate (EGCG) (Teavigo^®^ > 94%EGCG.) was purchased from Taiyo GmbH (Gevelsberg, Germany), and rat insulin ELISA kit was obtained from BtLAb (Huntington, NY, USA). Fasting blood glucose measurements of the rats were performed using PlusMED Accuro (Bionime Corporation, Taiwan, China) brand biosensor glucose meter and strips.

### 2.4. Statistical Evaluations—Data Analysis

Statistical Package for Social Science (SPSS) 16 package program was used. The results were given as mean ± standard deviation. “*t*-Test” was used to evaluate the relationship between numerical variables. The presence of statistically significant differences between the groups was analyzed by one-way Anova analysis.

## 3. Results

The data obtained as a result of the measurements performed on the control group and the groups given different doses of EGCG (50 mg/kg, 100 mg/kg, 200 mg/kg) are presented in Table 1. The data were taken at three different time periods (first day, 14th day, 21st day) and the mean values of each parameter are indicated. In addition, statistically significant differences between the groups were analyzed by one-way Anova. The places where significant differences were found were added to the table based on post hoc analysis (Dunnett).

On the first day, it was determined that there were no statistically significant differences between the groups in fasting blood glucose level, body weight, fasting insulin level, HOMA-IR, HOMA-IS, HOMA-B, and QUICK1 parameters (*p* > 0.05). In the measurements made on the first day of this study, there were no significant differences in metabolic parameters between the control group, 50 mg/kg, 100 mg/kg, and 200 mg/kg groups, indicating that all groups were equal in terms of these measurements.

The results of the 14th day of this study showed that there were significant differences in various biochemical parameters between the groups. Anova analysis of the fasting blood glucose levels showed statistically significant differences between the groups (*p* < 0.05). According to the results of the post hoc Dunnett analysis, significant differences were found between the control group (346.14) and the 100 mg/kg group (234.57) and 200 mg/kg group (192.86) (*p* < 0.05). On day 14, significant differences were found between the groups as a result of Anova analysis in terms of fasting insulin levels (*p* < 0.05). Post hoc Dunnett analysis showed that there were significant differences between the control group (5.63) and the 50 mg/kg group, 100 mg/kg group, and 200 mg/kg group (*p* < 0.05). On the day 14 HOMA-IR analysis, significant differences were found between the groups (*p* < 0.05). Significant differences were found between the control group (4.78) and the 50 mg/kg group (3.29), the 100 mg/kg group (2.06), and the 200 mg/kg group (3.42) (*p* < 0.05). Statistically significant differences were also observed in HOMA-IS and HOMA-B levels between the groups according to the results of Anova analysis (*p* < 0.05). When compared with the control group, HOMA-IS and HOMA-B levels were found to be statistically higher in the groups given EGCG (*p* < 0.05). On the 14th day, significant differences were found between the control group (0.30), 50 mg/kg group (0.32), and 100 mg/kg group (0.34) in terms of QUICKI levels (*p* < 0.05).

When the findings obtained as a result of the measurements performed on the 21st day of this study were analyzed, significant differences were found between the groups in terms of fasting blood glucose levels (*p* < 0.05). Statistically significant differences were found between the control group and 50 mg/kg group, 100 mg/kg group, and 200 mg/kg group (*p* < 0.05). Fasting blood glucose was significantly lower in the groups receiving EGCG (especially 200 mg/kg). On day 21, when analyzed in terms of body weight, statistically significant differences were found between the groups (*p* < 0.05). There were significant differences between the control group and the 200 mg/kg group. The body weight of the group receiving 200 mg/kg EGCG was significantly lower (*p* < 0.05). On day 21, the fasting insulin levels also showed significant differences between the groups (*p* < 0.05). Compared to the control group, fasting insulin levels increased statistically significantly in the groups given EGCG (*p* < 0.05). On day 21, significant differences were found between the groups in HOMA-IS and HOMA-B levels (*p* < 0.05). Compared to the control group, HOMA-IS and HOMA-B levels were statistically higher in the groups given EGCG (*p* < 0.05). On day 21, significant differences were also found between the groups in HOMA-IR and QUICKI levels (*p* < 0.05). Differences were found between the control group and 50 mg/kg group, 100 mg/kg group, and 200 mg/kg group (*p* < 0.05). Compared to the control group, the HOMA-IR levels decreased significantly and QUICKI levels increased significantly in the groups receiving EGCG.

Within the scope of this study, the results of the repeated measurements performed on the control group mg/kg, 100 mg/kg, and 200 mg/kg doses are presented below. Intragroup repeated measured comparisons of fasting blood glucose, body weight, fasting insulin, HOMA-IR, HOMA-IS, HOMA-B, and QUICKI parameters obtained within the scope of this study were performed by one-way analysis of variance (Anova) for repeated measures. For each parameter, the first day, 14th day, and 21st day data were given as mean ± standard error of the mean and statistical differences between groups were evaluated by the post hoc Bonferroni test. The findings obtained are given in Table 2.

When the repeated measurement results of the control group were analyzed, significant changes were observed in the metabolic parameters during the treatment period. Fasting blood glucose levels were 442.14 ± 28.04 mg/dL on the first day, 346.14 ± 16.60 mg/dL on day 14, and 328.00 ± 19.67 mg/dL on day 21. According to the post hoc Bonferroni test, a statistically significant difference was found between the first day and the 14th day (*p* = 0.022). Body weight increased over time. While the mean weight was 290.29 ± 7.72 g on the first day, it increased to 302.29 ± 7.66 g on day 14 and 317.14 ± 7.29 g on day 21. Statistically significant differences were found between the first day and the 14th day (*p* = 0.000), between the first day and the 21st day (*p* = 0.001), and between the 14th day and the 21st day (*p* = 0.005).

No significant difference was detected in fasting insulin levels. The value measured as 5.37 ± 0.22 μU/mL on the first day increased to 5.63 ± 0.20 μU/mL on day 14, but decreased to 5.06 ± 0.49 μU/mL on day 21. A decrease was observed in HOMA-IR (insulin resistance) values over time. While the mean was 5.83 ± 0.36 on the first day, it was 4.78 ± 0.17 on day 14 and 4.07 ± 0.40 on day 21. A significant difference was found between the first day and the 14th day (*p* = 0.040).

The HOMA-IS (insulin sensitivity) value showed an increase. The mean value was 4.32 ± 0.25 on the first day, 5.21 ± 0.19 on day 14, and 6.56 ± 0.82 on day 21, but these changes were not statistically significant. The HOMA-B values increased from 5.30 ± 0.49 on the first day to 7.38 ± 0.66 on day 14 and 7.16 ± 0.94 on day 21. The difference between the first day and the 14th day was statistically significant (*p* = 0.020). Finally, the QUICKI insulin sensitivity parameter was 0.30 ± 0.00 on the first day, 0.30 ± 0.00 on day 14, and 0.31 ± 0.01 on day 21. The difference between the first day and the 21st day was found to be significant (*p* = 0.049). 

Analyses based on repeated measurements of the 50 mg/kg dose group clearly demonstrated the effects of the treatment process on metabolic parameters. Fasting blood glucose levels decreased significantly as the treatment progressed. While the mean on the first day was 415.71 ± 19.95 mg/dL, it decreased to 304.43 ± 40.74 mg/dL on day 14 and 212.14 ± 18.73 mg/dL on day 21. According to the Bonferroni test results, this change was statistically significant both between the first day and the 14th day (*p* = 0.030) and between the first day and the 21st day (*p* = 0.001). Body weight values increased over time. The average body weight was 275.86 ± 8.95 g on the first day, 291.43 ± 10.93 g on the 14th day and 306.29 ± 12.08 g on the 21st day. According to post hoc analysis results, significant differences were found between the first day and the 21st day (*p* = 0.017) and between the 14th day and the 21st day (*p* = 0.044).

When fasting insulin levels were analyzed, a decrease was observed during the treatment period. While the mean value was 4.92 ± 0.26 μU/mL on the first day, this value decreased to 4.24 ± 0.30 μU/mL on day 21. There was a statistically significant difference between the first day and the 21st day (*p* = 0.010), which makes the decrease in the insulin levels significant. A significant decrease was also observed in terms of HOMA-IR values. HOMA-IR, which was measured as 5.06 ± 0.41 on the first day, decreased to 2.26 ± 0.29 on day 21. Statistically significant differences were found between the first day and day 14 (*p* = 0.033) and between the first day and day 21 (*p* = 0.001), indicating a significant decrease in insulin resistance.

The HOMA-IS parameter also showed a significant improvement throughout the treatment period. The value was 5.07 ± 0.40 on the first day and 12.50 ± 2.23 on day 21. There was a significant difference between the first day and the 21st day (*p* = 0.036), indicating a significant increase in insulin sensitivity. HOMA-B values also increased over time. While the mean value was 5.09 ± 0.35 on the first day, this value increased to 11.15 ± 1.30 on day 21. The difference between the first day and the 21st day was statistically significant (*p* = 0.013). Finally, the QUICKI insulin sensitivity parameter also showed statistically significant differences during the treatment period. The value of 0.30 ± 0.00 on the first day increased to 0.34 ± 0.01 on the 21st day and the difference between the first day and the 21st day was found to be significant (*p* = 0.003). These findings indicate that the 50 mg/kg dose has positive effects on metabolic parameters and provides significant improvements especially in fasting blood glucose, insulin resistance, and insulin sensitivity.

When the repeated measurements of the 100 mg/kg group were analyzed, significant changes were observed in various metabolic parameters over time. Fasting blood glucose levels showed a significant decrease during the treatment period. While the mean value was 370.00 ± 42.50 mg/dL on the first day, it decreased to 234.57 ± 11.16 mg/dL on day 14 and 179.43 ± 20.26 mg/dL on day 21. According to the post hoc Bonferroni test, statistically significant differences were found between the first day and the 14th day (*p* = 0.038), between the first day and the 21st day (*p* = 0.003), and between the 14th day and the 21st day (*p* = 0.026). There was no statistically significant difference in body weight.

Fasting insulin levels decreased during the treatment period. Insulin levels decreased from 5.38 ± 0.36 μU/mL on the first day to 3.58 ± 0.19 μU/mL on day 14 and 3.77 ± 0.21 μU/mL on day 21. There were significant differences between the first day and day 14 (*p* = 0.003) and between the first day and day 21 (*p* = 0.003). A significant decrease was also observed in HOMA-IR (insulin resistance) values. The HOMA-IR value, which was 4.93 ± 0.65 on the first day, decreased to 2.06 ± 0.12 on day 14 and 1.64 ± 0.16 on day 21. Statistically significant differences were found between day 1 and day 14 (*p* = 0.011) and between day 1 and day 21 (*p* = 0.003).

HOMA-IS (insulin sensitivity) values increased over time. Significant differences were found between the first day and day 14 (*p* = 0.004) and between the first day and day 21 (*p* = 0.001). HOMA-B values also increased during the treatment period. The mean value was 7.18 ± 1.32 on the first day, 7.75 ± 0.73 on day 14, and 14.11 ± 2.53 on day 21. A statistically significant difference was found between the first day and the 21st day (*p* = 0.048). Finally, the QUICKI parameter, another parameter evaluating insulin sensitivity, was 0.30 ± 0.01 on the first day, 0.34 ± 0.00 on day 14, and 0.36 ± 0.00 on day 21. Significant differences were found between the first day and the 14th day (*p* = 0.003) and between the first day and the 21st day (*p* = 0.000). These results indicate that there were improvements in fasting blood glucose, insulin resistance, and insulin sensitivity parameters in the 100 mg/kg dose group during the treatment period.

In the 200 mg/kg dose group, significant changes in metabolic parameters were observed during the treatment period. Fasting blood glucose levels showed a significant decrease during the treatment period. While the mean value was 410.57 ± 35.42 mg/dL on the first day, it decreased to 192.86 ± 11.51 mg/dL on day 14 and to 102.86 ± 4.63 mg/dL on day 21. According to the post hoc Bonferroni test results, statistically significant differences were found between the first day and the 14th day (*p* = 0.002), between the first day and the 21st day (*p* = 0.000), and between the 14th day and the 21st day (*p* = 0.000). Body weight decreased throughout the treatment period. The average body weight was 278.86 ± 7.74 g on the first day, 257.86 ± 5.44 g on day 14, and 244.57 ± 3.34 g on day 21. According to the results of statistical analysis, statistically significant differences were found between the first day and the 14th day (*p* = 0.000), between the first day and the 21st day (*p* = 0.001), and between the 14th day and the 21st day (*p* = 0.002)

Fasting insulin levels increased throughout the treatment period in this group. Statistically significant differences were found between day 1 and day 14 (*p* = 0.003), day 1 and day 21 (*p* = 0.002), and day 14 and day 21 (*p* = 0.040). The HOMA-IR (insulin resistance) parameter decreased throughout the treatment period. Significant differences were found between the first day and day 14 (*p* = 0.034), between the first day and day 21 (*p* = 0.003), and between day 14 and day 21 (*p* = 0.004).

The HOMA-IS (insulin sensitivity) value increased during treatment. Significant differences were found between the first day and day 14 (*p* = 0.008), between the first day and day 21 (*p* = 0.000), and between day 14 and day 21 (*p* = 0.016). A dramatic increase was also observed in the HOMA-B parameter. Similarly, significant differences were found between day 1 and day 14 (*p* = 0.007), between day 1 and day 21 (*p* = 0.000), and between day 14 and day 21 (*p* = 0.000). Finally, the QUICKI parameter, another indicator of insulin sensitivity, was 0.30 ± 0.00 on day 1, 0.32 ± 0.00 on day 14, and 0.33 ± 0.00 on day 21. A significant difference was found between the first day and the 21st day (*p* = 0.005). These results indicate that in the 200 mg/kg dose group, there were significant improvements in metabolic parameters such as fasting blood glucose, insulin resistance, and insulin sensitivity as well as a decrease in body weight during the treatment period.

**Figure 2:**
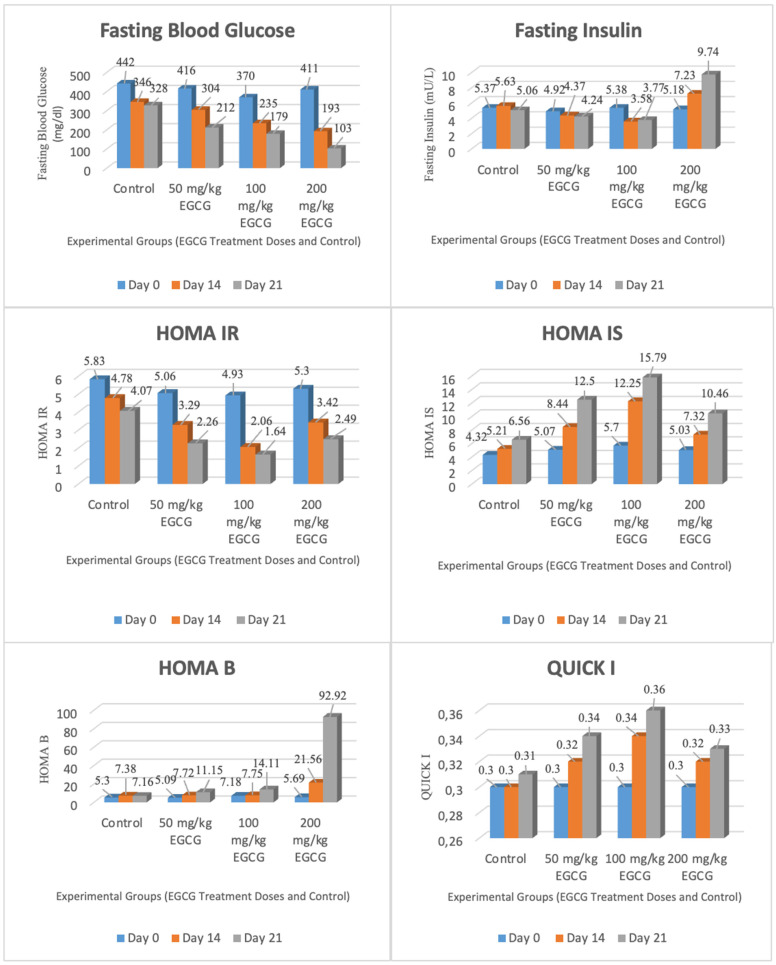
Comparison of diabetes and insulin resistance parameters with repeated measurements.

Figure 2 demonstrates how the fasting glucose levels, body mass, fasting insulin concentrations, HOMA-IR, HOMA-IS, HOMA-B, and QUICKI metrics changed across three time points (day 0, day 14, and day 21) in the control and various dose groups (50 mg/kg EGCG, 100 mg/kg EGCG, 200 mg/kg EGCG).

## 4. Discussion

The adverse effects of urbanization, the adoption of modern lifestyles, poor dietary habits, sedentary lifestyles, increased stress, and surrounding conditions all play a crucial role in the alarming rise of diabetes worldwide [40]. Despite recommendations for behavioral adjustments and medical therapies to prevent this condition, these measures have not been effective in reducing the rising incidence [41]. Therefore, recently, the issue of finding alternative natural substances in the prevention and treatment of diabetes has attracted interest and has been investigated. Studies on the subject have revealed the microbe-fighting, free radical scavenging, and virus-fighting properties of polyphenols [42]. EGCG has been proven to be a powerful antioxidant and protects cell membranes from oxidative stress. EGCG, the major polyphenol found in green tea, is well known for its wide range of health benefits [43], primarily due to its phenolic hydroxyl groups [44].

In this study, the aim was to evaluate the potential benefits of EGCG in an STZ-induced diabetic rat model. The STZ-induced diabetes model successfully mimics the key features of diabetes, such as insulin resistance and hyperglycemia, and therefore provides a suitable model for this study [45].

When the literature was reviewed, it was reported that intraperitoneal EGCG administration decreased hyperglycemia and partially maintained islet mass in STZ-induced diabetic mice [46] and also reduced blood glucose in both Sprague Dawley and also Zucker rats [47]. When the literature is examined, the results of studies examining the effects of oral EGCG on diabetes are contradictory. In mice fed a high-fat diet (HFD), there was no blood glucose-lowering effect after 4 weeks, but in type 2 diabetic mice, EGCG administration can improve glucose tolerance in vivo [48]. This suggests that EGCG may be effective under certain conditions and that certain types of diet may limit these effects. HFD may negatively affect glucose metabolism by increasing insulin resistance, which may reduce the potential benefits of EGCG. Therefore, the efficacy of EGCG may vary depending on the dietary habits and current health status of individuals. In our study, analyses based on the results of repeated measurements of the 50 mg/kg dose group clearly revealed the effects of the treatment process on fasting blood glucose. A significant reduction in fasting blood glucose levels was noted as the treatment progressed. When the results of another study were analyzed, it showed that an EGCG supplementation of 100 mg per kg orally for 2 weeks via gavage significantly improved type 2 diabetes mellitus in preclinical models. Consistent with our study [49], when repeated measurements of the 100 mg/kg group were analyzed, significant changes were observed in various metabolic parameters over time. Fasting blood glucose levels showed a significant decrease during the treatment period. The observed decrease in fasting blood glucose over time is believed to be associated with enhanced insulin sensitivity or improvements in glucose regulation.

Body weight is considered a key risk factor in the onset of various persistent illnesses, including diabetes. Excess body weight can contribute to heightened insulin resistance and irregularities in glucose metabolism. Therefore, control of body weight plays a critical role in the mitigation and management of diabetes and other chronic diseases [50]. EGCG has potential health benefits in body weight management. EGCG has been reported to markedly reduce lipid accumulation in fat-storing cells, can increase AMPK activity, and promote mitochondrial functions as well as increase the expression of thermogenic genes. These effects promote the conversion of white fat cells to brown fat cells by inhibiting adipogenesis [51]. Under the action of EGCG, AMPK pathways are activated and a significant decrease in triglyceride levels in preadipocytes is observed, which triggers lipid degradation in adipocytes [52].

In the literature, many studies with similar dosages have examined the effects of EGCG on body weight. For instance, after 17 weeks of EGCG treatment, crucial reductions in weight increase, mesenteric adipose tissue buildup, fasting blood glucose, insulin resistance, serum cholesterol levels, and fatty liver severity were noted in obese mice [53]. In our study, there were statistically significant differences between the control and treatment groups in terms of body weight. 

Insulin resistance and decreased Beta cell quantity and function have been proven by many studies to be the main and main cause of T2DM [54,55]. In this study, we showed that EGCG significantly reduced fasting insulin levels in STZ-induced diabetic rats. This is in parallel with the results of this study using similar doses in the literature. Lower fasting insulin levels indicated an improvement in insulin sensitivity and potentially a reduction in insulin resistance [56,57]. The antioxidant properties and anti-inflammatory effects of EGCG may have positive effects on biochemical pathways that increase insulin release and regulate glucose metabolism [54]. These findings emphasize the potential of EGCG in the management of metabolic syndrome and also diabetes and increase the clinical importance of EGCG as a dietary supplement. 

HOMA-IR, the ultimate criterion for the assessment of insulin resistance, was also used in our study and the HOMA-IR values of rats were calculated [58]. Besides HOMA-IR, other mathematical models analyzing B-cell function were also applied for a more comprehensive evaluation of insulin resistance. These models include HOMA-B, quantitative insulin sensitivity index (QUICKI), and HOMA-IS [54]. As a result of our study, a decrease in HOMA-IR values was observed, the most significant decrease was observed in the 100 mg/kg group and HOMA-B values increased over time. In the 200 mg/kg group, this increase was most pronounced. HOMA-IS values increased over time. The most significant increase was observed in the 100 mg/kg group. In the 100 mg/kg EGCG and 200 mg/kg EGCG groups, the QUICK I values of rats were significantly increased. The results obtained reveal the positive effects of EGCG on insulin resistance and show its potential to increase insulin sensitivity. When the literature is examined, the results of the existing studies are in parallel with our study findings [59,60]. In a rat study, administration of the oral antidiabetic drug (metformin) and EGCG (50 and 100 mg/kg) caused an increase in both the number and density of beta cells, suggesting that both treatments have similar and comparable effects in promoting the repair of islet beta cells in the treated group [54].

These findings suggest that EGCG may help control diabetes by reducing insulin resistance and may be an effective component in diabetes management, especially at high doses. Doses of 100 mg/kg and 200 mg/kg stood out as the most effective doses in terms of their effects on insulin resistance.

The EGCG doses of 50, 100, and 200 mg/kg used in our study were transformed into human doses by the Reagan–Shaw method and calculated as 8.1 mg/kg, 16.2 mg/kg, and 32.4 mg/kg, respectively [61]. These doses are considered achievable for humans; however, for higher doses, EGCG supplementation may be considered, provided that its safety is thoroughly assessed. The human equivalent doses obtained by the Reagan–Shaw method provide an important basis for predicting the potential effects of the effects observed in rats on humans. In line with these data, the applicability of the recommended doses of EGCG for humans should be discussed and the safety and efficacy of these doses should be further investigated in clinical trials.

## 5. Conclusions

In conclusion, the positive effects of EGCG on diabetes and insulin resistance were clearly demonstrated in this study, proving that EGCG may be a promising therapeutic modality in diabetes management and reductions in insulin resistance. Thus, the inclusion of EGCG in medical nutrition therapy would contribute to achieving glycemic control and improving insulin sensitivity in individuals with diabetes. Clinical studies are needed to clearly define and demonstrate the feasibility and efficacy of these effects in human populations, as well as to determine effective doses, and need to be validated in human clinical trials.

## Figures and Tables

**Table 1:** Comparison of parameters obtained at different time periods in the research groups.

Variable	First Day (Mean ± SE)				Day 14 (Mean ± SE)				Day 21 (Mean ± SE)			
	Control	50 mg/kg	100 mg/kg	200 mg/kg	Control	50 mg/kg	100 mg/kg	200 mg/kg	Control	50 mg/kg	100 mg/kg	200 mg/kg
Fasting blood glucose (mg/dL)	442 ± 28	416 ± 20	370 ± 43	411 ± 35	346 ± 17	304 ± 41	235 ± 11	193 ± 12	328 ± 20	212 ± 19	179 ± 20	103 ± 5
Post Hoc Dunnett	None				A with C (*p* = 0.007); A with D (*p* = 0.000)				A and B (*p* = 0.000); A and C (*p* = 0.000); A and D (*p* = 0.001)			
Body weight (G)	290 ± 8	276 ± 9	267 ± 8	279 ± 8	303 ± 8	291 ± 11	286 ± 16	258 ± 5	317 ± 7	306 ± 12	298 ± 15	245 ± 3
Post Hoc Dunnett	None				None				A with D (*p* = 0.000)			
Fasting insulin (mU/L)	5.37 ± 0.22	4.92 ± 0.26	5.38 ± 0.36	5.18 ± 0.24	5.63 ± 0.20	4.37 ± 0.32	3.58 ± 0.19	7.23 ± 0.18	5.06 ± 0.49	4.24 ± 0.30	3.77 ± 0.21	9.74 ± 0.70
Post Hoc Dunnett	None				A and B (*p* = 0.002); A and C (*p* = 0.000); A and D (*p* = 0.000)				A with D (*p* = 0.000)			
HOMA-IR	5.83 ± 0.36	5.06 ± 0.41	4.93 ± 0.65	5.30 ± 0.61	4.78 ± 0.17	3.29 ± 0.56	2.06 ± 0.12	3.42 ± 0.16	4.07 ± 0.40	2.26 ± 0.29	1.64 ± 0.16	2.49 ± 0.25
Post Hoc Dunnett	None				A and B (*p* = 0.007); A and C (*p* = 0.000); A and D (*p* = 0.014)				A and B (*p* = 0.001); A and C (*p* = 0.000); A and D (*p* = 0.002)			
HOMA-IS	4.32 ± 0.25	5.07 ± 0.40	5.70 ± 0.91	5.03 ± 0.56	5.21 ± 0.19	8.44 ± 0.94	12.25 ± 0.87	7.32 ± 0.37	6.56 ± 0.82	12.50 ± 2.23	15.79 ± 1.25	10.46 ± 0.94
Post Hoc Dunnett	None				A and B (*p* = 0.007); A and C (*p* = 0.000)				A with B (*p* = 0.018); A with C (*p* = 0.000)			
HOMA-B Cell	5.30 ± 0.49	5.09 ± 0.35	7.18 ± 1.32	5.69 ± 0.63	7.38 ± 0.66	7.72 ± 1.42	7.75 ± 0.73	21.56 ± 3.00	7.16 ± 0.94	11.15 ± 1.30	14.11 ± 2.53	92.92 ± 8.64
Post Hoc Dunnett	None				A with D (*p* = 0.000)				A with D (*p* = 0.000)			
QUICKI	0.30 ± 0.00	0.30 ± 0.00	0.30 ± 0.01	0.30 ± 0.00	0.30 ± 0.00	0.32 ± 0.01	0.34 ± 0.00	0.32 ± 0.00	0.31 ± 0.01	0.34 ± 0.01	0.36 ± 0.00	0.33 ± 0.00
Post Hoc Dunnett	None				A and B (*p* = 0.005); A and C (*p* = 0.000)				A and B (*p* = 0.003); A and C (*p* = 0.000); A and D (*p* = 0.022)			

A: Control; B: 50 mg/kg; C: 100 mg/kg; D: 200 mg/kg.

**Table 2:** Repeated measurement results for all groups.

Variable	Control (Mean ± SE)			50 mg/kg (Mean ± SE)			100 mg/kg (Mean ± SE)			200 mg/kg (Mean ± SE)		
	İlk Gün	14. Gün	21. Gün	İlk Gün	14. Gün	21. Gün	İlk Gün	14. Gün	21. Gün	İlk Gün	14. Gün	21. Gün
Fasting Blood Glucose (mg/dL)	442 ± 28	346 ± 17	328 ± 20	416 ± 20	304 ± 41	212 ± 19	370 ± 43	235 ± 11	179 ± 20	411 ± 35	193 ± 12	103 ± 5
Post Hoc Bonferroni	A with B (*p* = 0.022)			A with B (*p* = 0.030); A with C (*p* = 0.001)			A ile B (*p* = 0.038); A ile C (*p* = 0.003); B ile C (*p* = 0.026)			A with B (*p* = 0.002); A with C (*p* = 0.000); B with C (*p* = 0.000)		
Body Weight (G)	290 ± 8	302 ± 8	317 ± 7	276 ± 9	291 ± 10	306 ± 12	267 ± 8	286 ± 16	298 ± 15	279 ± 8	258 ± 5	245 ± 3
Post Hoc Bonferroni	A with B (*p* = 0.000); A with C (*p* = 0.001); B with C (*p* = 0.005)			A with C (*p* = 0.017); B with C (*p* = 0.044)			None			A with B (*p* = 0.000); A with C (*p* = 0.001); B with C (*p* = 0.002)		
Fasting Insulin (mU/L)	5.37 ± 0.22	5.63 ± 0.20	5.06 ± 0.49	4.92 ± 0.26	4.37 ± 0.32	4.24 ± 0.30	5.38 ± 0.36	3.58 ± 0.19	3.77 ± 0.21	5.18 ± 0.24	7.23 ± 0.18	9.74 ± 0.70
Post Hoc Bonferroni	None			A with C (*p* = 0.010)			A with B (*p* = 0.003); A with C (*p* = 0.003)			A with B (*p* = 0.003); A with C (*p* = 0.002); B with C (*p* = 0.040)		
HOMA-IR	5.83 ± 0.36	4.78 ± 0.17	4.07 ± 0.40	5.06 ± 0.41	3.29 ± 0.56	2.26 ± 0.29	4.93 ± 0.65	2.06 ± 0.12	1.64 ± 0.16	5.30 ± 0.61	3.42 ± 0.16	2.49 ± 0.25
Post Hoc Bonferroni	A with B (*p* = 0.040)			A with B (*p* = 0.033); A with C (*p* = 0.001)			A and B (*p* = 0.011); A and C (*p* = 0.003)			A with B (*p* = 0.034); A with C (*p* = 0.003); B with C (*p* = 0.004)		
HOMA-IS	4.32 ± 0.25	5.21 ± 0.19	6.56 ± 0.82	5.07 ± 0.40	8.44 ± 0.94	12.50 ± 2.23	5.70 ± 0.91	12.25 ± 0.87	15.79 ± 1.25	5.03 ± 0.56	7.32 ± 0.37	10.46 ± 0.94
Post Hoc Bonferroni	None			A and B (*p* = 0.019); A and C (*p* = 0.036)			A with B (*p* = 0.004); A with C (*p* = 0.001)			A with B (*p* = 0.008); A with C (*p* = 0.000); B with C (*p* = 0.016)		
HOMA-B	5.30 ± 0.49	7.38 ± 0.66	7.16 ± 0.94	5.09 ± 0.35	7.72 ± 1.42	11.15 ± 1.30	7.18 ± 1.32	7.75 ± 0.73	14.11 ± 2.53	5.69 ± 0.63	21.56 ± 3.00	92.92 ± 8.64
Post Hoc Bonferroni	A with B (*p* = 0.020)			A with C (*p* = 0.013)			A with C (*p* = 0.048)			A with B (*p* = 0.007); A with C (*p* = 0.000); B with C (*p* = 0.000)		
QUICKI	0.30 ± 0.00	0.30 ± 0.00	0.31 ± 0.01	0.30 ± 0.00	0.32 ± 0.01	0.34 ± 0.01	0.30 ± 0.01	0.34 ± 0.00	0.36 ± 0.00	0.30 ± 0.00	0.32 ± 0.00	0.33 ± 0.00
Post Hoc Bonferroni	None			A and B (*p* = 0.020); A and C (*p* = 0.003)			A and B (*p* = 0.003); A and C (*p* = 0.000)			A with C (*p* = 0.005)		

A: first day (day 0), B: day 14, and C: day 21.

## Data Availability

The data that support the findings of this study will be made available by the corresponding author upon reasonable request. Data are not publicly available due to privacy and ethical concerns.
